# Outcomes of an Alpha-DC-1 Dendritic Cell-Based Vaccine Clinical Trial in Patients with Low-Tumor-Burden High-Risk Ovarian Carcinoma

**DOI:** 10.3390/cancers18081285

**Published:** 2026-04-18

**Authors:** Patrick J. Stiff, Cheryl M. Czerlanis, Ronald K. Potkul, Margaret Liotta, Zheng Yu, Lori Pease, Swarnali Banerjee, Swati Mehrotra, Abigail Winder, Jennifer Guevara, Diane Palmer, Maureen L. Drakes

**Affiliations:** 1 Department of Medicine, Cardinal Bernardin Cancer Center, Stritch School of Medicine, Loyola University Chicago, Building 112, 2160 South First Avenue, Maywood, IL 60153, USA; pstiff@lumc.edu (P.J.S.); cczerlanis@medicine.wisc.edu (C.M.C.); 2 Department of Obstetrics and Gynecology, Cardinal Bernardin Cancer Center, Loyola University Chicago, 2160 South First Avenue, Maywood, IL 60153, USA; rpotkul@lumc.edu (R.K.P.); mliotta@lumc.edu (M.L.); abigail.winder@luhs.org (A.W.); 3 Cellular Therapy Center, Cardinal Bernardin Cancer Center, Loyola University Medical Center, 2160 South First Avenue, Maywood, IL 60153, USA; zhyu@coh.org (Z.Y.);; 4 Center for Data Science and Consulting, Department of Mathematics and Statistics, Loyola University Chicago, Chicago, IL 60660, USA; sbanerjee@luc.edu; 5 Department of Pathology, Loyola University Medical Center, Building 110, 2160 South First Avenue, Maywood, IL 60153, USA; swati.mehrotra@va.gov; 6 Cancer Clinical Trials Office, Cardinal Bernardin Cancer Center, Loyola University Chicago, 2160 South First Avenue, Maywood, IL 60153, USA; jguevara@luc.edu (J.G.);

**Keywords:** ovarian cancer, dendritic cell vaccines, tumor microenvironment, progression-free survival, overall survival, platinum sensitivity status

## Abstract

**Background/Objectives:** High-grade serous ovarian cancer (HGSOC) is usually discovered in advanced stages and often relapses shortly after initial conventional therapy. Survival in HGSOC patients might be improved with the use of novel immune therapies, which potentiate autologous anti-tumor responses. Dendritic cells (DCs) are potent antigen-presenting cells that can initiate immune responses, activate cytotoxic T cells and drive T-cell differentiation. This pilot trial evaluated the safety and efficacy of a unique DC vaccine (α-DC-1) in relapsed, advanced HGSOC patients with minimal tumor burden. **Methods:** Monocytes from patient leukaphereses were used to propagate a unique autologous DC, the α-DC-1, generated with granulocyte–macrophage colony-stimulating factor and interleukin-4, pulsed with keyhole limpet hemocyanin (KLH) and tumor lysate (from debulking surgery) on day 5, and matured with a cocktail of cytokines and chemokines on day 6. Mature α-DC-1 were harvested on day 7 and administered intranodally (inguinal nodes) every other week for three doses/cycle for up to three DC vaccine cycles (nine vaccines). The primary endpoints were progression-free survival (PFS) and overall survival (OS). **Results:** In 19 patients treated, the median PFS was 9.7 months (95% CI: (5, NA)) and the median OS was 42.2 months (95% CI: (31.2, 68.3)). In 5/19 (26.3%) patients, OS exceeded five years. Administration of six or more vaccines was associated with a significant improvement in PFS. No grade 2 or higher toxicities were noted. **Conclusions:** Our α-DC-1 vaccine was safe, and 94.2% elicited an immune response to KLH. The long OS, exceeding 5 years in some patients, suggests this DC vaccine may improve survival for some with relapsed HGSOC.

## Simple Summary

In patients with high-grade serous ovarian cancer (HGSOC), surgical debulking with subsequent platinum- and taxane-based combination chemotherapy is initially effective treatment; however, most patients relapse and soon die. Survival might be improved with the use of novel immune therapies, which boost autologous anti-tumor responses. Dendritic cells (DC) are potent antigen-presenting cells that activate cytotoxic T cells to fight cancer. This trial evaluated the safety and efficacy of a unique autologous mature DC vaccine (alpha-DC-1) administered intranodally to relapsed, advanced HGSOC patients. In 19 patients treated, the median progression-free survival (PFS) was 9.7 months (95% CI: (5, NA)), and the median overall survival (OS) was 42.2 months (95% CI: (31.2, 68.3)). In 26% of these patients, OS exceeded five years. Administration of six or more vaccines was associated with a significant improvement in PFS. The long OS in some patients suggests this DC vaccine may improve survival for some HGSOC patients.

## 1. Introduction

In the United States, there will be about 21,010 new cases of ovarian cancer diagnosed and 12,450 deaths in 2026 [1]. High-grade serous ovarian carcinoma (HGSOC), the most common and aggressive subtype of epithelial ovarian cancer, is characterized by late-stage diagnosis, marked therapy resistance, and high recurrence rates. First-line therapy of surgical debulking with subsequent platinum- and taxane-based combination chemotherapy is initially effective treatment; however, most patients relapse and eventually die [2,3,4,5,6].

Due to the current poor outcomes in patients with HGSOC, there is a critical need to develop new therapies. Recently, there have been significant improvements in progression-free (PFS) and overall survival (OS) in relapsed platinum-resistant disease with treatments including the antibody–drug conjugate, mirvetuximab soravtansine-gynx [7], and the glucocorticoid receptor antagonist, relacorilant with nab-paclitaxel [8]. Several clinical trials have also tested immunotherapies in an effort to improve prognosis. Initial trials focused on testing anti-programmed death-1 (anti-PD-1) antibodies in refractory ovarian cancer, agents which have produced survival improvements in many other solid cancers [9,10,11] but initially proved to be of little benefit in the treatment of ovarian cancer [12,13,14,15,16,17]. This lack of efficacy has been attributed to the low numbers of immunocompetent immune infiltrating T cells and high numbers of immune-inhibitory cells, among other factors, in the ovarian tumor microenvironment (TME), which actively suppresses immune responses [18,19]. However, the very recent phase III KEYNOTE B-96 study, which tested pembrolizumab and paclitaxel ± bevacizumab, indicated both an improved PFS and OS for platinum-resistant disease with an impressive 4-month OS improvement [20]. Thus, we see that new strategies that incorporate immunotherapies are promising in the management of this disease.

Our approach to relapsed/refractory ovarian cancer therapy was to investigate a novel dendritic cell vaccine, using autologous tumor cells collected from either the primary or relapsed tumor. Dendritic cells (DCs) are potent antigen-presenting cells (APCs) that activate autologous effector CD4 and CD8 T cells [21,22]. In the normal ovarian tumor microenvironment (TME), these events are not efficient due to the sparseness of DCs as well as their diminished function [23,24]. Treatments that can circumvent immune suppression and restore DC function could potentially induce T cells to generate anti-tumor specific immunity [25,26,27,28]. Our hypothesis was that a series of nine alpha dendritic cell-1 (α-DC-1) vaccines to autologous tumor antigens would be safe and change the local tumor microenvironment (TME) such that there would be an autologous anti-tumor response that may affect survival after vaccination.

DC clinical trials have previously been conducted in ovarian cancer utilizing several approaches for modifying DCs prior to vaccines [29,30,31,32,33,34]. Even though cancer DC vaccines are generally regarded as safe, exhibiting limited grade 2 or higher toxicities, to date, there has been no monumental success in phase III DC trials; thus, efforts to maximize this therapy outcome are still needed.

This pilot study examined the safety (primary endpoint) and efficacy (secondary endpoint) as measured by progression-free survival (PFS) and overall survival (OS) of an autologous tumor/mature alpha dendritic cell-1 (α-DC-1) vaccine [35] administered intranodally [36,37] to HGSOC patients. This report primarily focuses on the relationship between the number of vaccine doses administered to patients and disease outcome. The study endpoint was PFS and OS as measured from the time of first vaccine administration in each patient. Study results showed that DC vaccine therapy was safe, induced anti-tumor immune responses and that a higher number of vaccine doses correlated with increased PFS.

## 2. Materials and Methods

### 2.1. Trial Design

Subjects were enrolled in this phase II pilot clinical trial of an autologous α-DC-1 vaccine at Loyola University Health System (LUHS) between 2011 and 2017. The protocol and consent documents were approved by the Loyola University Institutional Review Board (IRB). The primary goal was to establish the safety of this novel autologous α-DC-1 vaccine in ovarian cancer and to obtain preliminary evidence of efficacy in patients with relapsed/refractory ovarian cancer with minimal residual disease (MRD) after re-debulking surgery to obtain the viable tumor needed for vaccine preparation. Subsequent salvage chemotherapy was administered if an MRD state was not established by the surgical procedure.

Eligibility included patients who failed to enter complete remission with induction therapy or relapsed after a period of remission and were not eligible for otherwise curative therapy. Patients who underwent primary suboptimal surgical debulking followed by induction chemotherapy and were in a < complete response (CR), who were believed to be at high risk of rapidly developing recurrent disease, were also eligible. Candidates were referred either locally from our practice or through phone/email inquiries from patients/physicians who obtained protocol information from the Clinical Trials.gov website. This latter group was first evaluated distantly via phone interview by a research nurse, and if believed to be eligible, this was followed by an on-site evaluation.

For the initial phase of the protocol, all candidates needed to have an accessible tumor as demonstrated by examination of CT scans, to obtain tumor cells for vaccine preparation. In addition, as the goal was to establish an MRD state post-operation, patients needed to have such a disease state pre-operation as evaluated by the surgical team. Other eligibility criteria included no anti-neoplastic chemotherapy, radiotherapy or immunotherapy for four weeks preceding protocol entry. Additional inclusion criteria included a life expectancy of >3 months, a Karnofsky performance status >70% and a WBC >2500/mm^3^, a platelet count >80,000/mm^3^, normal renal and hepatic function and a lymphocyte count >500/mm^3^. Exclusion criteria included uncontrolled intercurrent illness including, but not limited to, ongoing or active infection; active bleeding; symptomatic congestive heart failure; unstable angina pectoris; cardiac arrhythmia; uncontrolled bronchospasm; hypertension, hyperglycemia, or hypercalcemia; or psychiatric illness/social situations that in the opinion of the investigators would compromise the patient’s ability to tolerate this treatment or affect compliance. Patients with HIV infection, AIDS, or hepatitis B surface antigen positivity were also excluded from this trial. Patients on combination antiretroviral therapy were ineligible because of the potential for pharmacokinetic interactions. Patients with a history of corticosteroid use in the four weeks preceding entry into the clinical trial, or the requirement of ongoing corticosteroid use during the study period, and patients requiring therapeutic anticoagulation during the trial period, or with known brain metastases were also excluded. Patient histological grade and stage were characterized by a pathologist on formalin-fixed paraffin-embedded (FFPE) tissue sections.

If eligible for the vaccine trial and after giving written informed consent, patients underwent a surgical re-debulking procedure performed by our Gynecologic Oncology team with the goal to establish an MRD state (maximum tumor diameter < 1 cm in maximal diameter). Tumors were collected under sterile conditions and transferred to our cGMP facility for collagenase digestion, tumor cell preparation and evaluation as described below. Patients proceeded to the vaccination portion of the study if sufficient tumor cells were collected for a minimum of three vaccines, and the patient was in an MRD state. If patients were not in an MRD state, they were required to undergo salvage chemotherapy to establish MRD before vaccination. Once ready for vaccination, the autologous tumor lysate (TL) was used to pulse autologous monocyte-derived DCs, and the α-DC-1 vaccine [35] was administered intranodally [37] in the patients’ inguinal lymph nodes, under ultrasound guidance by an attending physician in the Radiology treatment suite. Subjects received 3 doses each cycle for up to three vaccine cycles (9 doses), unless limited by disease progression or insufficient yield of tumor lysate (TL) for the pulsing of subsequent doses of the DC vaccine. De-identified data was collected by the Cancer Center Clinical Trials Office research nurse and compiled into a protocol report. Toxicities were graded according to the National Cancer Institute Common Terminology Criteria for Adverse Events, version 4.0.

### 2.2. Dendritic Cell Vaccine Preparation and Characterization

**Figure 1:**
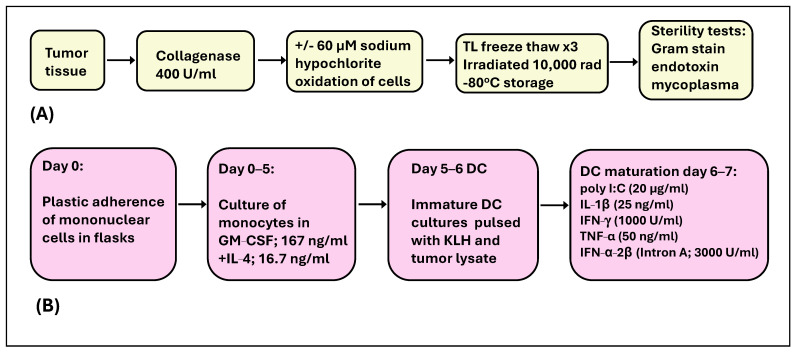
Preparation of dendritic cells for vaccine administration. Tumor lysate (TL) was prepared from excised ovarian tumors by collagenase digestion (Panel (**A**)). Monocyte-derived dendritic cells were generated from patient leukapheresis cells in GM-CSF and IL-4 over 5 days. Immature DCs were pulsed with KLH (150 µg/mL) + 1–1.5 × 10^6^ tumor cells equivalent/mL of lysate/25 cm^2^ flask (days 5–6). From days 6–7, DCs were matured in a cocktail of soluble molecules (Panel (**B**)). On day 7, mature α-DC-1 were harvested and tested for sterility.

A portion of the excised tumor was sent to Loyola University Department of Pathology for histology and pathology assessment to confirm disease diagnosis. Autologous tumor cell lysate was prepared from another portion of the tumor tissue under current Good Manufacturing Practices (cGMP) conditions (Figure 1A) for the pulsing of DCs generated from each patient. To yield sufficient tumor cells for the pulsing of DCs, tumor masses of greater than 2 × 2 cm in diameter were obtained at the time of surgery. Preparation of single-cell suspensions of the tumor was performed by collagenase digestion (400 U/mL; Catalog No. C5138; Sigma, St. Louis, MO, USA). Cells obtained from collagenase-digested tumor tissue were oxidized by sodium hypochlorite treatment (60 µM in PBS) or left untreated. Single-cell suspensions of tumor lysate were snap freeze–thawed (liquid nitrogen 120 s, followed by a 37 °C water bath for 5 min incubation) three times in rapid succession and then irradiated with 10,000 rad in an irradiator (^137^Cs irradiator, LUHS). The tumor lysate was stored at −80 °C for later pulsing of DCs. Cytospins of tumor lysate were prepared for the pathological evaluation of malignant cells to quantify the number of tumor cells in the patient sample. Tumor lysate was tested for infectious agent contamination with culture and Gram stains, as well as for the presence of endotoxins (Limulus Amebocyte Lysate-Kinetic QCL kit; Lonza, MD, USA) and mycoplasma (Roche Diagnostics, Indianapolis, IN, USA) according to the manufacturer’s instructions.

Dendritic cell vaccines were prepared in a cGMP facility. Briefly, the patient leukapheresis (LK) product was enriched for mononuclear cells on a Ficoll-Hypaque gradient. This fraction was cryopreserved for the propagation of autologous DC vaccines for each dose, as scheduled for patients. LK cells were thawed, and monocytes were isolated by the adherence of mononuclear cells on plastic 75 cm^2^ flasks in a 37 °C, 5% CO_2_ humidified incubator for 2–3 h. Monocytes were cultured in granulocyte–macrophage colony-stimulating factor (GM-CSF; 167 ng/mL, Sanofi, Morristown, NJ, USA) and interleukin-4 (IL-4,16.7 ng/mL, R&D Systems, Inc. Minneapolis, MN, USA) for 5 days for the propagation of immature DC.

DC were loaded with tumor lysate (TL) and pulsed with KLH (IMMUCOTHEL^®^, Biosyn, Carlsbad, CA, USA) [38,39] in culture on day 5. Briefly, on day 5 of DC culture in GM-CSF and IL-4, 5–6.5 × 10^6^ DC were transferred to 25 cm^2^ flasks and pulsed with freeze–thawed autologous tumor cell lysate (1–1.5 × 10^6^ tumor cell equivalents/mL of lysate for each flask) and KLH (150 μg/mL). DCs were matured by the addition of a cocktail of soluble molecules, consisting of poly I:C (20 µg/mL, Invivogen, San Diego, CA, USA), IL-1β (25 ng/mL, CellGenix, Portsmouth, NH, USA), IFN-γ (1000 U/mL, R&D Systems), TNF-α (50 ng/mL, CellGenix), and IFN-α-2β (Intron A; 3000 U/mL, Schering Corporation, Kenilworth, NJ, USA), to cultures from day 6 to 7 (Figure 1B) [35,40]. The resulting mature DCs were harvested on day 7 of culture.

Cells were evaluated for typical DC morphology (veiled appearance by phase microscopy) and cell viability by trypan blue exclusion. DC products for injection had a viability of >70%. To ensure sterility of the vaccine, aliquots of day 5 and day 7 culture samples were tested for the presence of endotoxin and mycoplasma, a Gram stain was also performed, and the aliquots were cultured for bacteria and fungus by the Loyola Clinical Microbiology Laboratory for a period of 14 days. Prior to administering to patients, phenotypic characterization was done for each DC vaccine preparation for all patients. This was performed by monoclonal antibody staining, flow cytometry and the acquisition of events on a flow cytometer (Becton Dickinson FACS CANTO II machine, San Jose, CA, USA) to verify that, among other characteristics, the cell population exhibited high-cell-surface CD1a, HLA-DR (MHC class II), CD40, CD80, CD83, and CD86 expression, and other surface markers associated with mature DCs [35,40].

DCs were resuspended in 2 mL PBS and injected into the inguinal lymph nodes of patients under ultrasound guidance with vaccine doses ranging from 0.4 to 13.8 × 10^6^ CD1a+ DCs. Patients received a maximum of 9 vaccines, administered on days 0, 14 and 28 per cycle for a total of three cycles, unless there were limitations, such as patients exhibiting disease progression or insufficient yield of TL for the pulsing of subsequent doses of the DC vaccine. Three vaccines were given per cycle, with an interval of 4 weeks between cycles.

### 2.3. Evaluation of Clinical Outcomes

Patients had a baseline CT scan before the start of treatment. A CT scan was done prior to each vaccine cycle, then approximately every three months until progression for up to 2 years. Disease assessments for progression were performed utilizing CT scans and CA-125 levels [41]. In the event of a raised CA125 level, progression was confirmed by imaging. Patients were monitored for local and/or systemic reactions, such as fever or chills, low blood pressure, and nausea and/or vomiting after each vaccine was administered.

Delayed-type hypersensitivity (DTH) skin testing responses to the autologous tumor lysate and KLH were evaluated at about day 61 after the first vaccine administration of each cycle. Patients were injected in the left arm with 0.1 mL of 2 µg, 20 µg and 200 µg KLH in 100 µL PBS or with TL in the right arm. Erythema and induration were measured 48 h later.

### 2.4. Immune Profiling Before Treatment

The TME of patients at the time of surgery was investigated by immunohistochemistry (IHC) antibody staining of FFPE ovarian tumor tissue using primary antibodies for CD3 (F7.2.38; 1:1000 dilution, Dako, Glostrup, Denmark), CD8 (C8/144B; 1:100 dilution, Cell Marque, Rocklin, CA, USA) and FoxP3 (236A/E7; ab 20034, Abcam, Waltham, MA, USA). Sections were incubated in a biotinylated secondary antibody for peroxidase (PK 6102, Vector Laboratories, Burlingame, CA, USA), followed by an avidin–biotin–peroxidase complex and enzyme reagent (ABC, Vector Laboratories). Sections were developed in Vector NovaRED (SK4800) or diaminobenzidine (DAB; SK4100, Vector Laboratories), counterstained in hematoxylin and mounted in a Vectamount H-5000 (Vector Laboratories). Pathology scoring was performed by a graded system from 0 to 4, where 4 was the highest frequency of stained cells.

To further understand events in the ovarian TME and survival outcome, a pilot study was conducted by imaging mass cytometry (IMC; Standard BioTools, Markham, Ontario, Canada), a procedure that detects over 30 cell types/cell markers simultaneously in FFPE tissue [42,43]. The antibody panel used for this staining is described in Supplemental Table S2. Imaging was done using CyTOF software v9 on the Hyperion XTi Imaging System. For this pilot study, patients were selected who had high, medium or low OS intervals after DC vaccine therapy. The human tonsil was used as a positive control, and polycystic ovarian disease FFPE tissue was used as a negative control. Preview Mode whole-slide subsampling was used to guide the selection of single-cell resolution regions of interest (ROIs) in immune-/T-cell-rich regions. Two ROIs in each tissue were selected for viewing by MCD Viewer.

Analysis was conducted with the goal of cell phenotyping and visualization for each sample, with emphasis on T cells. Samples were analyzed on a Visiopharm 2024.07x 64 platform, and downstream analysis was done in R4.4.1. By Visiopharm, a cell segmentation algorithm was developed to extract all marker intensities and positivity within each cell. A pre-trained deep learning algorithm was used to detect nuclei using the Ir191/193 DNA channels, followed by the dilation of the cell body from the individual cells. By downstream analysis, an R Script was developed for phenotyping of each cell. Thresholds were set on a marker-by-marker basis, applied to all cells, and phenotypes were designated in an unsupervised manner using dominant marker analysis to resolve ambiguity and subclassify phenotypes. Visualization was done with XY plots, heat maps and dimensionality reduction with UMAPs.

### 2.5. Immune Profiling After Treatment

For in vitro studies to evaluate clinical outcomes, 30cc of blood was removed from patients at frequent intervals, including before and after each vaccine administration. Peripheral blood mononuclear cells (PBMCs) were prepared on a Ficoll-Hypaque gradient. Enzyme-linked immunosorbent (ELISpot; 1-DIK3420-1000; Mabtech, Cincinnati, OH, USA) assays were set up to evaluate tumor-specific T-cell activity from pre- and post-DC vaccine therapy (day 61) for each patient. Briefly, PBMCs collected at leukapheresis, and at the DTH (day 61) time point were stimulated with phorbol myristate acetate (PMA, 50 ng/mL; Sigma Chemical Co., St. Louis, MO, USA) and ionomycin (250 ng/mL; Tocris Bioscience, Ellisville, MO, USA) as a positive control, or challenged with KLH (40 µg/mL) or tumor lysate (3–5 µL/100 µL volume in wells). IFN-γ-secreting spots were detected using an Avidin Biotin Enzyme complex kit (Vectastain ABC kit) and a peroxidase substrate (AEC kit, Vector Laboratories, Burlingame, CA, USA). Spots were developed for 10 min, then the plates were washed and air-dried. To measure whether there was an increase in IFN-γ-secreting cells in the culture after a 48 h assay, the number of IFN-γ-secreting spots on ELISpot plates was captured on a C.T.L Cellular Technology Immunospot machine and evaluated by using Immunospot 3 and 5.0.3 software (Cleveland, OH, USA), respectively.

The frequency of several immune cell types, including T regulatory cells (CD4+CD25highFoxP3+CD127low), CD4+ T, CD3+ T and CD8+ T cells, was routinely studied in patient PBMCs at pre- and post-vaccine administration time points (in monthly blood draws) by monoclonal antibody staining and acquisition of events on a flow cytometer (BD Canto II, San Jose, CA, USA). The antibody panel used included mouse anti-human antibodies (BD Pharmingen, San Diego, CA, USA) for surface staining as follows: CD4 PerCP (BD 550631), CD25 PECy7 (BD 557741), and CD127 Alexa Fluor 647 (BD 558588). Nuclear staining of cells in the same tube was performed using PE-anti-human FoxP3 (Cat. # 320108, clone 206D, Biolegend, San Diego, CA, USA) and True Nuclear Transcription Buffer Set (Cat. # 424401; Biolegend, San Diego, CA, USA), according to the manufacturer’s instructions. Data were analyzed using the FlowJo program (Ashland, OR, USA).

Additionally, for each patient, PBMCs were stained with monoclonal antibodies conjugated to multiple fluorochromes to identify the surface expression of molecules, including CD3, CD8, CD28, CD45RO (all on T cells), CD19 (B cells), CD40 (co-stimulatory molecules), CD14 (macrophages) and other antigens on immune cells, before and after patients were administered DC vaccines. Different tubes were set up for staining using 2 or more antibodies in each tube and the following antibodies: CD3 FITC (BD 555339), CD8 FITC (BD 555634), CD28 PE (BD 555729), CD45RO APC (BD 559865), CD19 PE (BD 555413), CD40 APC (BD 555501) and CD14 FITC (BD 555397). Events were acquired on a flow cytometer (BD Canto II), and data were analyzed using the FlowJo program (version 8.8.6).

### 2.6. Statistical Analysis

Overall survival and progression-free survival probabilities were estimated and were graphed using Kaplan–Meier plots. Each survival curve was accompanied by a 95% confidence region. Median OS and median PFS were estimated using Kaplan–Meier survival estimates. Logrank tests were performed to determine significant differences in survival in patients who received varying doses of vaccines. Tests were performed using age as a continuous variable and again as a categorical variable (categories “average or below”, “above average”). The number of vaccines was used as a numerical variable and again as a categorical variable (“6 or higher” or “less than 6”). Cox proportional hazards models were fit to test the interaction effect between and the main effect of several parameters, including age and number of vaccines. All analyses and visualizations were done in R [44]. Significance levels are indicated as *p*-value < 0.05.

## 3. Results

### 3.1. Patients and Clinical Outcome

Overall, 48 patients were entered into the initial surgical debulking step. Of these, 19 patients were entered into step 2 and were administered at least one dose of vaccine. Patient clinical characteristics are shown in Table 1. Of the remaining 29 (of 48) patients, 18 had insufficient tumor for a minimum of three vaccines to proceed with vaccination; 9 did not respond to post-surgical debulking chemotherapy to achieve a MRD state, in one patient the tumor specimen was contaminated, and one declined to proceed to vaccination after responding to salvage chemotherapy. Patient recruitment was concluded in 2017, and this final study evaluation was done starting in 2024, after the completion of the in vitro assays and a sufficient follow-up period to more accurately determine long-term OS. As all patients vaccinated were in a low-tumor-burden state after surgical re-debulking or salvage chemotherapy, a response rate to this therapy could not be measured. Of the cohort of 19 study participants, 17 were diagnosed with HGSOC, one was diagnosed with poorly differentiated malignant tumor with sarcomatoid features, and one was diagnosed with poorly differentiated mixed endometroid and transitional type carcinoma. Twelve of 19 patients (63%) were platinum-sensitive, and 7 of 19 (37%) patients were platinum-resistant. Many of the platinum-sensitive patients were in their third or greater remission.

At the time of diagnosis, patients were between 39.7 and 74.7 years of age (average 56.1 years +/− s.d. 8.87). Of the 19 patients enrolled, 2 patients (11%) received 2 vaccines, 3 (16%) received 3 vaccines, 4 (26%) received 6 vaccines, 2 (11%) received 8 vaccines, and 8 (37%) received all 9 vaccines. All DC vaccines administered to patients were characterized for cell surface markers, as shown in Table 2. As shown for the representative phenotypic characterization pattern for the six DC vaccines administered to patient 60 ML, these cells expressed high levels of cell surface CD1a and CD11c, which are typical DC markers; high levels of costimulatory molecules CD40, CD80 and CD86 (mature DC markers); and almost all DCs expressed HLA-DR (MHC-class II). These α-DC-1 generally had a high expression of the chemokine receptor CCR7, a molecule characteristic of mature DCs [45].

Table 1 summarizes the number of vaccines each patient received and PFS and OS after the first vaccine. The median PFS after the first vaccine dose was 9.7 months (95% CI: (5, NA)), and the median OS was 42.2 months (95% CI: (31.2, 68.3)). There was no significant difference in PFS or OS between platinum-sensitive vs. platinum-resistant patients. There were no serious vaccine-related adverse events observed in the cohort (i.e., no grade 2 or greater toxicities) and no protocol deviations.

### 3.2. Evaluation of Patient Outcome by Number of Vaccine Doses

**Figure 2:**
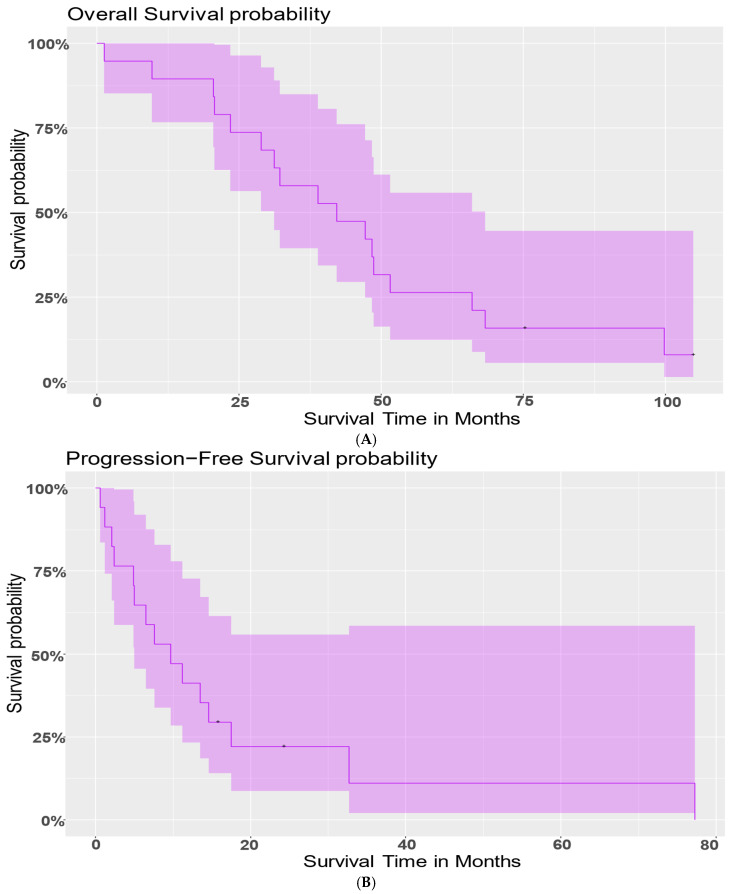
Patient survival outcome. Figures show survival curves for (**A**) overall survival in DC vaccine patients and (**B**) progression-free survival in DC vaccine patients. In the patient cohort, the median progression-free survival was 9.7 months (95% CI: (5, NA)), and the median overall survival was 42.2 months (95% CI: (31.2, 68.3)).

To understand whether the number of vaccines administered to patients correlated with survival, a Cox proportional hazards model was fit to model the risk of death (OS) with the number of vaccines patients received. The number of vaccines was statistically significant with a *p*-value of 0.0383 (Figure 2A). Additionally, the estimated hazard ratio between a patient with one more vaccine than another was 0.812. This indicates that a patient with one extra vaccine had 81.21% risk of death compared to another; that is, with one additional vaccine, the model shows an almost 20% reduced risk of death, and therefore, longer survival.

Similarly, as per the Cox proportional hazards model, the number of vaccines was statistically significant (*p*-value = 0.0011) for risk of progression (Figure 2B). The estimated hazard ratio = 0.5972 indicates that with one additional vaccine, a person would have a 59.72% risk of progression compared to another patient. Thus, an additional vaccine reduced the risk of progression by 40.28%.

**Figure 3:**
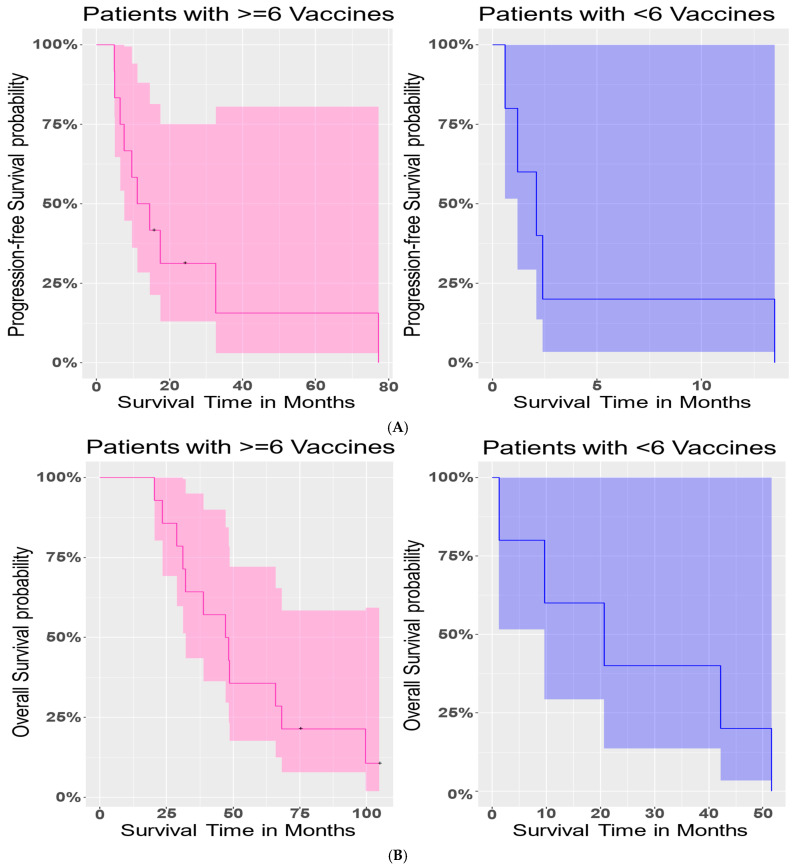
Survival outcome in patients with greater than 6 vaccine doses. Plot (**A**) shows progression-free survival in patients with 6 or more vaccines vs. patients with 2–5 vaccines. There was a significant difference in PFS (*p*-value = 0.003) between these two sub-groups of patients. Plot (**B**) represents overall survival in patients with 6 or more vaccines vs. patients with 2–5 vaccines. At the 5% level, no significant difference in overall survival (*p*-value = 0.06) was observed.

To further determine how survival varied with the administration of vaccine cycles, we investigated PFS and OS outcomes in patients who received six vaccines or more (2–3 cycles) compared with patients who received fewer than two full vaccine cycles. There was a significant difference in PFS (Logrank test, *p*-value = 0.003) between these two sub-groups of patients (Figure 3A). Patients who had six or more vaccines had a longer PFS interval. In contrast, however, there was no significant difference in OS (*p*-value = 0.06) between these two sub-groups of patients (Figure 3B).

Next, we considered whether these differences between patients with six vaccines or more and those with fewer than six vaccines were affected by age. The average age of patients was 56.08 years. We grouped patients as “average or below” and “above average.” We had 9 patients whose age was average or below, and 10 patients who were above average. The interaction effect between the number of vaccines and age was not significant (*p*-value = 0.0600) in a Cox-PH model. We found that age by itself did not have a significant effect (*p*-value = 0.9400), and upon adjusting for age groups, the number of vaccines was no longer significant (*p*-value = 0.0956) for OS. Similar analysis for PFS revealed no significant interaction effect (*p*-value = 0.241) between age groups and number of vaccines. While age groups were still not significant (*p*-value = 0.7183), the number of vaccines had a significant effect (*p*-value = 0.0061) on PFS. The estimated risk of progression in patients with six or more vaccines was only 71.12% of that in patients with fewer than six vaccines. Thus, patients with six or more vaccines had almost a 29% lower chance of progression than those with fewer than six vaccines.

**Figure 4:**
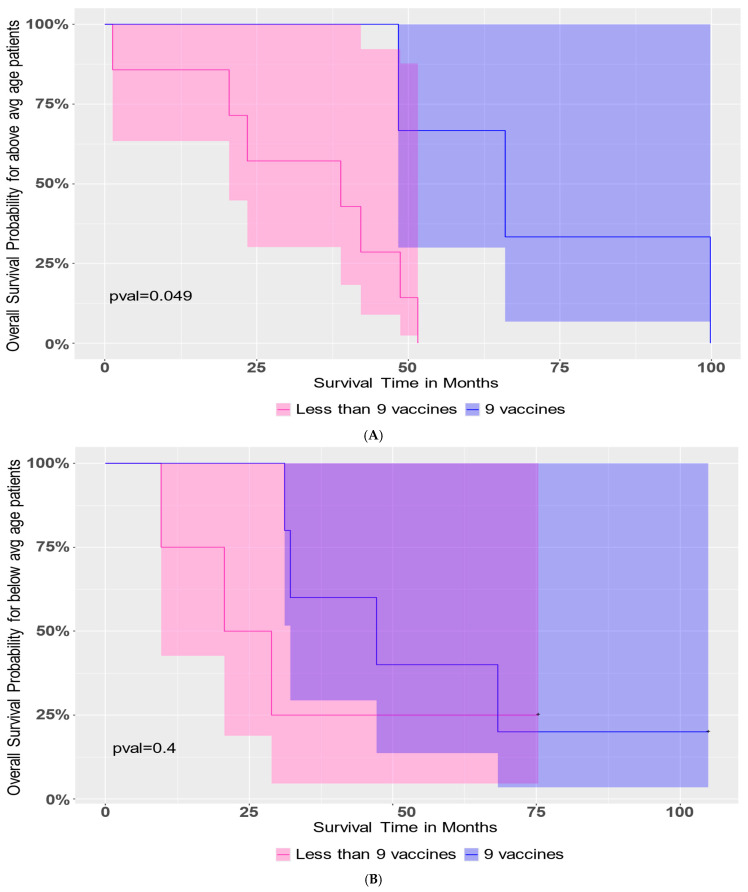
Survival outcome in older patients receiving 9 vaccines. Patients were stratified into two groups, above-average (avg) age and below-average age, at the time of diagnosis. Tests were performed using age as a continuous variable and again as a categorical variable (categories “average or below”, “above average”). The number of vaccines was used as a numerical variable and again as a categorical variable (“6 or higher” or “less than 6”). Cox proportional hazards models were fit to test the interaction effect between and the main effect of several parameters, including age and number of vaccines. Plots show Kaplan–Meier survival curves with 95% confidence regions. A Logrank test showed that older patients who received 9 vaccines (Panel (**A**), *p*-value = 0.049) had better overall survival than older patients who received fewer than 9 vaccines (Panel (**B**), *p*-value = 0.4).

We studied whether the administration of nine vaccine doses was beneficial to PFS or OS in patients, along with the consideration of age stratification. Nine vaccines led to an improvement in OS in patients who were above-average age (56.1 years) at the time of diagnosis (*p*-value = 0.049, Figure 4A). This trend was not observed in the below-average age patient group (Figure 4B). There was no significant difference in PFS for either age stratum of patients who received nine vaccines.

### 3.3. Patient Tumor Microenvironment at the Time of Diagnosis

The immune activity of the TME in patients at the time of diagnosis may influence the survival of patients [46,47,48,49,50]. To address this, we studied FoxP3 T regulatory cells, CD3 and CD8 T cells in FFPE ovarian tumors of several patients by IHC. Pathology scores (0–4) for each antigen were summarized (Supplemental Table S1). These results gave a broad context of tumor-infiltrating lymphocyte (TIL) distribution across patient ovarian tissues. It was observed that TILs scattered in the tumor tissue were often seen in clusters or, in some patient tissues, in a scattered pattern.

**Figure 5:**
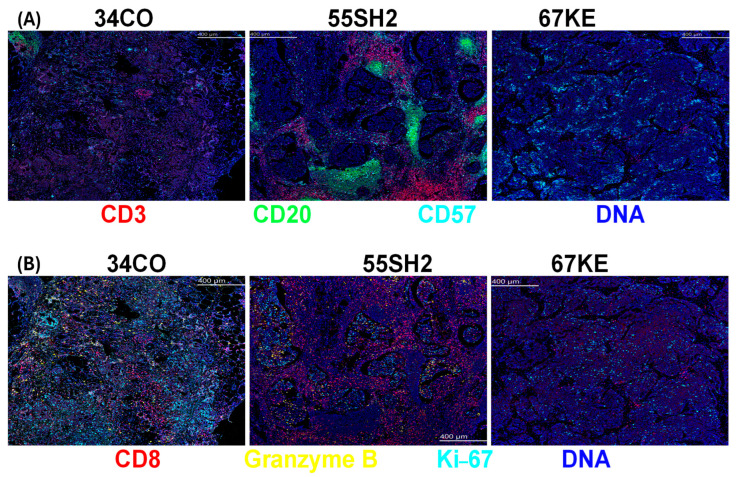
Differential characterization of patient tumor by imaging mass cytometry. FFPE tumors were studied to identify the expression of several phenotypic and functional markers by imaging mass cytometry (IMC; Standard BioTools). Results are shown for CD3 (red), CD20 (green), CD57 (aquamarine) and DNA (blue) in Panel (**A**). Results are shown for CD8 (red), granzyme B (GrB, yellow), Ki67 (aquamarine) and DNA (blue) in Panel (**B**) for patients 34CO, 55SH2 and 67KE. Panels show greater enrichment of immune cells in patient 55SH2 than in patient 34CO or patient 67KE.

For the further evaluation of patient TME and potential impact on patient survival, we selected patients with different lengths of OS for IMC staining for a pilot study, focusing primarily on cell types that are associated with immune reactivity. The full list of antibodies used for IMC is shown in Supplemental Table S2. As shown for selected cell subsets, spatial images for IMC-stained ovarian FFPE sections revealed that patient 67KE (0.8 yr OS) has lower CD3, CD8, granzyme B, Ki67 and CD20 (B cell) levels than patient 34CO (4.1 yr OS), who has less positively stained cells for these markers than patient 55SH2 (most abundant in these immune cells; 8.5 yr OS) (Figure 5A,B).

**Figure 6:**
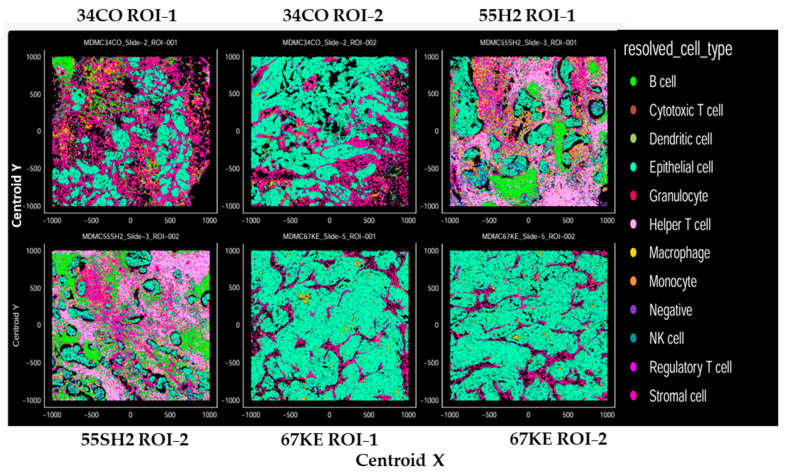
Supervised cell phenotyping in patient tumor tissue. Dominant marker analysis was used to determine phenotypes in each sample. XY plot panels display the resulting spatial output after dominant marker analysis was applied to cells. Cells were plotted by centroid X and centroid Y coordinates and colored by supervised phenotypic output. Plots are shown for 2 ROIs in the tumor tissue sample, each from patients 34CO, 55SH2 and 67KE.

In further support of the observations above, phenotyping by dominant marker analysis showed that the TME of patient 55SH2 was abundant in helper CD4 T cells, B cells, cytotoxic CD8 T cells and macrophages. The TME of patient 34CO was enriched in macrophages and had a high distribution of stromal and epithelial cells. Patient 67KE showed a deficit of CD4 helper T cells and CD8 cytotoxic T cells, whereas there was a very high proportion of epithelial cells (Figure 6).

As per downstream analysis, the numerical density of cell phenotypes in two ROIs of each patient sample stained by IMC was determined. The studies also used an ovarian negative control (non-malignant sample) and tonsil FFPE tissue (positive control). The values shown in Table 3 represent the percentage of abundance of stained cells for each marker or group of markers relative to the total number of cells. The category shown as negative represents cells that did not stain for any of the cell markers studied, whereas NA indicates that there were no cells of that type in that sample ROI.

Consistent with the MCD Viewer images (Figure 5A,B), the sample from patient 55SH2 was more abundant in B cells, CD8+ T cells and CD4+ T cells than that from patient CD34CO, followed by patient 67KE. The average cellular density across two ROIs for T cells (CD3, CD4 and CD8) was 36, 1.95 and 0.64 for patients 55SH2, 34CO and 67KE, respectively. This suggests that the TME in patient 55SH2 (with the longest post-vaccine survival of the three patients studied) was enriched in critical immune cells in comparison with patient 34CO and patient 67KE. Additional evaluation showed that patient 55SH2 had the least amount of epithelial cells, with average cell density over two ROIs of 11.34, 54 and 85.64 for 55SH2, 34CO and 67KE, respectively (Table 3).

### 3.4. Patient In Vivo and In Vitro Immune Responses

DTH skin testing was performed for a majority of patients who had at least one full cycle of vaccines administered (excluding patients 67KE, 63MM2 and 80WS). A positive DTH response to KLH was reported in 15/16 (93.7%) patients. On the contrary, a positive DTH response to tumor lysate was only observed in 1/16 patients (patient 70VS).

A positive ELISpot response was determined to be more than twice the number of IFN-γ spots in treatment wells in comparison with the number of spots in the control medium. ELISpots were conducted for all patients at LK and at post-vaccine time points similar to Figure S1. Studies analyzing PBMCs obtained from DC vaccine patients after one or more cycles of vaccine administration at the DTH time point showed that 16/17 (94.2%) patients tested (excluding 67KE and 63MM2) resulted in positive ELISpots for KLH (patient 52MK was negative). On the other hand, a positive ELISpot response for IFN-γ secretion of PBMCs obtained at the DTH time point and stimulated with TL was observed in 2/17 (11.8%) patients. Patients 40KR and 46KK had a positive IFN-γ response for PBMCs obtained at the DTH time point when stimulated with TL.

**Figure 7:**
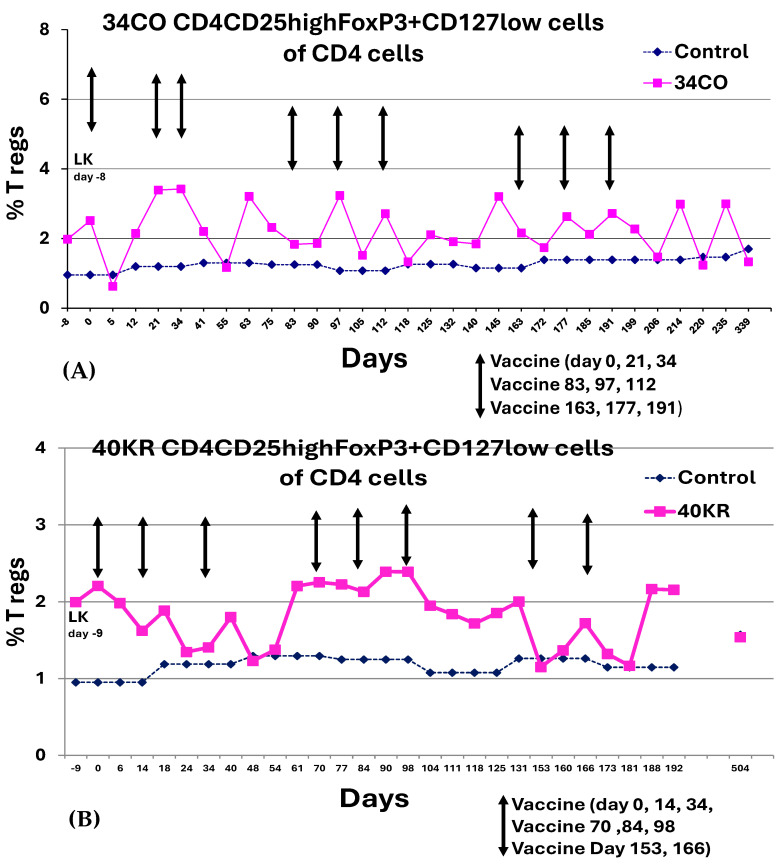
FoxP3 expression in PBMCs of DC vaccine patients. PBMCs collected at the time points indicated were surface-stained with anti-human antibodies (BD Pharmingen, San Diego, CA, USA) using CD4 PerCP (BD 550631), CD25 PECy7 (BD 557741), and CD127 Alexa Fluor 647 (BD 558588). Cells were then fixed and permeabilized, and nuclear staining of cells was performed with PE-anti-human FoxP3 (Cat. # 320108, clone 206D, Biolegend, San Diego, CA, USA) and True Nuclear Transcription Buffer Set (Cat. # 424401; Biolegend). Events were acquired on a flow cytometer; gating was done in the lymphocyte region, and data were analyzed using the FlowJo 8.8.6 program. Representative dot plot analysis is summarized for (**A**) patient 34CO and (**B**) patient 40KR. The times of vaccine administration are indicated by arrows.

T regulatory cells in study patients were evaluated by analyzing flow cytometry data to determine CD4+CD25highFoxP3+CD127low cells in the CD4 T-lymphocyte population. Changes in FoxP3 T regulatory cell expression with vaccine administration revealed oscillating patterns, as shown below, with no definitive trends coinciding with treatment. Representative data is shown in Figure 7A,B. PBMC phenotypes characterized by flow cytometry post-DC vaccine administration did not reveal any notable trends in percent changes in CD3, CD4, CD8 T-cell subsets, macrophages or B cells with treatment (Table S3).

## 4. Discussion

Ovarian cancer is a lethal gynecologic cancer worldwide [51]. In HGSOC, surgical debulking and subsequent platinum- and taxane-based combination chemotherapy are initially effective treatments, but the majority of patients become resistant to this therapy, leading to poor outcomes [3,4,5,6]. Recent studies have shown modest but significant improvements in OS for platinum-resistant disease [7,8,20], but the elimination of MRD in patients who have relapsed remains elusive. DC pulsed with tumor antigens are capable of inducing a potent immune response and have been tested in clinical trials [30]. The goal of DC-based vaccines is to stimulate the patient’s own immune system, especially in a low-tumor-burden state, as observed in our patients [25,26,27]. Furthermore, such anti-tumor immune responses may generate immunological memory, which could delay or prevent disease relapse.

We investigated α-DC-1 cells, which are high-IL-12p70-secreting DCs and are capable of generating a potent effect on TILs inside the tumor, potentially leading to potent anti-tumor effects [35,52]. The primary study endpoint of our study was the ability to safely administer nine vaccines, intranodally in proximity to the tumor location, over an extended period of time, with secondary endpoints of PFS and OS measured from the time of first vaccine administration. Exploratory endpoints included immune responses to the vaccine, both in vivo and in vitro. We found no grade 2 or greater toxicities from the administration of the vaccines. While PFS was relatively short for this subset of patients, their median OS of 42 months was longer than we expected. In addition, we found that the number of vaccine doses administered was an important factor in patient outcomes, such that with one additional vaccine, the Cox proportional hazards model showed an almost 20% reduced risk of death (i.e., longer survival in patients). The administration of six or more vaccines significantly favored PFS and resulted in a modest but insignificant improvement in OS. Nine vaccines led to an improvement in overall survival in patients who were above-average age at the time of diagnosis, but not in those who were below average.

A similar finding of prolonged OS but with a rather brief PFS post-vaccine administration, as observed in our patient cohort, was reported previously with an FDA-approved prostate cancer vaccine. In this double-blind placebo-controlled multi-center phase III clinical trial (NCT00065442) for metastatic castration-resistant prostate cancer, 314 patients were assigned Sipuleucel-T and 171 patients were assigned a placebo [53]. The Sipuleucel-T vaccine consisted of autologous PBMCs, including APCs (DCs) that were activated ex vivo with a recombinant fusion protein, PA2024 (a prostate antigen, prostatic acid phosphatase fused to GM-CSF). The results showed that in the Sipuleucel-T group, there was a 4.1-month improvement in median survival (25.8 months in the Sipuleucel-T group and 21.7 months for the group receiving placebo), but no difference in PFS. The 36-month survival probability was 31.7% in the Sipuleucel-T group, while only 23.0% in the placebo group [53]. The OS improvement may be due to the fact that the immune effects caused by the vaccine led to an improved response to subsequent therapies administered at relapse.

Other reports strongly support the efficacy of DC vaccines in this disease. For example, in one ovarian cancer study (NCT02107937, EudraCT2010-021462-30) [29], autologous dendritic cells for DCVAC were differentiated from patients’ CD14^+^ monocytes, pulsed with two allogenic ovarian cancer cell lines (SK-OV-3, OV-90) and matured in the presence of polyinosinic: polycytidylic acid (p-I:C) (DCVAC/OvCa). This DCVAC/OvCa given after chemotherapy was associated with a statistically significant improvement of PFS in patients undergoing first-line treatment of epithelial ovarian cancer (FIGO III) [29].

This novel α-DC-1 vaccine has also shown efficacy in a glioma phase II trial, having a therapeutic effect on survival when used in combination with the standard regimen chemoradiotherapy (consisting of 60 Gy plus concurrent temozolomide) [54].

In our clinical trial, greater than 90% of patients mounted an immune response to KLH in DTH testing and by ELISpot for IFN-γ; however, only a few patients showed an immune response to TL. The latter observation may suggest that, in our study, whereas TL may provide a broad battery of tumor-associated antigens, it may not be robust enough to provoke an immune response to TL in most patients, due to the lack of concentration of any single specific tumor antigen.

There are limitations to our study, including the small number of pre-selected patients ultimately eligible for vaccination (19/48 enrolled to step 2), whose survival was expected to be longer than that of a typical relapsed ovarian cancer patient. Thus, no firm conclusions can be made based on our median survival of 42.2 months, as the majority of relapsing patients did respond to subsequent salvage therapy. A randomized trial would therefore be needed to verify efficacy. In addition, though enrolled patients were primarily in the broad category of high-grade serous ovarian carcinoma, each patient had a different immune status and history at the time of diagnosis, which might have regulated the response to immune therapy. Importantly, the TME of each patient was vastly different, as shown by the in-depth TME study for patients 34CO, 55SH2 and 67KE by IMC, where there was an abundance of B cells, CD8+ T cells and CD4+ T cells in patient 55SH2. Downstream analysis further showed that several subsets of immune cells were enriched in the TME of patient 55SH2, while there was a distinct contrast with patient 67KE, which primarily showed an epithelial cell phenotype. In a separate study using FFPE samples and staining by IHC, the H score for patient CD8 T-cell expression for patient 55SH2 was 150 (a high H-score), whereas the H score for CD8 for patient 67 KE was 56 (a low H-score), confirming the sparsity of CD8 T cells in the TME of patient 67KE. These TME observations and the fact that patient 55SH2 had an overall survival that was several-fold longer than that of patient 67KE support the idea that patient 55SH2 exhibited a TME immune contexture that was conducive to a more favorable survival outcome. These data support the current thinking that tumor cellular characteristics impact patient outcomes [48].

## 5. Conclusions

HGSOC is often a lethal malignancy. The administration of an α-DC-1 vaccine intranodally to HGSOC patients showed a significant PFS benefit in patients who received a higher number of doses. Furthermore, nine vaccines led to an improvement in overall survival in patients who were above-average age at the time of diagnosis. Study outcomes suggest that α-DC-1 vaccine administration may be effective in improving OS. However, as this was a select population, i.e., MRD after surgical debulking at relapse ± salvage chemotherapy, it will be necessary to do a randomized trial to determine efficacy.

DC vaccines may boost anti-tumor immune responses and could be combined with other immunotherapies, such as an anti-PD-1 antibody, or with targeted therapies. Such combinations may further stimulate the immune system to attack cancer cells, which may lead to improved outcomes in HGSOC patients.

## Figures and Tables

**Table 1:** Patient characteristics, progression-free survival (PFS) and overall survival (OS) of ovarian cancer DC vaccine trial cohort.

PATIENT I.D.	No. of Vaccines	Age at Time of Diagnosis (Years)	Platinum Sensitivity	PFS from 1st Vaccine (Months)	Overall Survival from 1st Vaccine (Months)	OS from 1st Vaccine (Years)
63MM2	2	56.4	resistant	2.4	42.2	3.6
67KE	2	53.3	resistant	0.6	9.7	0.8
48JS	3	53.6	sensitive	2.1	20.7	1.8
69NW	3	57.0	sensitive	13.5	51.6	4.4
80WS	3	74.7	resistant	1.2	1.3	0.1
52MK	6	58.1	resistant	5.0	38.9	3.3
60ML	6	66.0	sensitive	4.9	23.5	2.0
78LC2	6	62.3	sensitive	11.2	20.5	1.7
79SP2	6	65.3	sensitive	32.7	48.7	4.1
40KR	8	51.2	resistant	17.5	28.9	2.5
88MLM	8	39.7	sensitive	24.3	A (75.3)	6.4 (A)
34CO	9	60.5	sensitive	N/A *	48.4	4.1
46KK	9	49.8	resistant	14.6	31.2	2.7
55SH2	9	69.5	sensitive	77.3	99.8	8.5
59EDC	9	48.4	sensitive	N/A *	32.2	2.7
66TS	9	45.8	sensitive	7.6	47.2	4.0
57LMO	9	50.2	sensitive	15.8	A (104.9)	8.9 (A)
70VS	9	57.5	resistant	9.7	66.0	5.6
83SF	9	46.3	sensitive	6.5	68.3	5.8

“A” indicates the patient was alive at the time of the last census. * N/A indicates there was no evidence of disease progression. Platinum-sensitive patients were those who relapsed > 6 months after primary therapy, and platinum-resistant patients were those who relapsed within 6 months of completing primary therapy, as well as those in second or greater relapse.

**Table 2:** Phenotypes of injected vaccines.

Cell Phenotype	Vaccine 1	Vaccine 2	Vaccine 3	Vaccine 4	Vaccine 5	Vaccine 6
CD1a	65.5	57.8	66.6	69.4	72.3	72.8
CD11c	79.9	78.5	89.6	88.1	87.7	55.1
CD40	82.8	81.5	91.1	90.1	88.0	88.4
CD80	74.5	71.7	85.4	83.7	82.7	79.5
CD83	51.2	49.9	67.0	60.1	63.6	68.5
CD86	85.9	86.7	93.4	93.8	92.5	91.7
HLA-DR	98.2	97.7	99.0	99.1	98.8	98.9
CCR7	68.6	67.2	78.3	75.3	71.2	N.D.

Each day 7 α-DC-1 vaccine product for all patients was characterized for surface phenotypes by monoclonal antibody staining and flow cytometry. DCs were stained in several tubes with antibodies, including CD1a APC (BD Pharmingen 559775), HLA-DR FITC (BD 555811), CD40 APC (BD 551073), CD80 FITC (BD 555683), CD83 APC (BD 551073), CD86 (BD 555680), CD11c PE (BD 555392) and CCR7 FITC (BD561271), using appropriate isotype IgG controls. Events were acquired on a flow cytometer. In FlowJo analysis, gating was done in the live cell region, and the dot plots were summarized. Table 2 shows the percentage of cells in quadrants positive for each cell antigen identified. The table shown is for the α-DC-1 phenotypes of the six vaccines administered to patient 60 ML and is representative of the phenotypic characterization done for all DC vaccines for all patients. N.D. indicates not done.

**Table 3:** Cellular Density of Resolved Phenotypes. The density of resolved phenotypes by sample is tabulated. Values represent the percentage abundance relative to the total number of cells. Cell density is normalized to the total cellular count of each sample. Values are shown for 2 ROIs for each ovarian cancer patient tissue sample and 1 ROI for ovarian benign tissue (OVCON; negative control) and for tonsil tissue (TON; positive control) samples. NA indicates that there were no cells of that cell subset detected.

Cell Type	34CO-ROI-1	34CO-ROI-2	55SH2-ROI-1	5SH2-ROI-2	67KE-ROI-1	67KE-ROI-2	OVCON-ROI-1	TON-ROI-1
B cell (CD20)	7.71764	0.04620	13.1106	21.34535	NA	NA	0.00352	32.72244
Cytotoxic T cell (CD8)	1.92549	0.23434	5.06134	3.84530	0.23963	0.23040	0.27507	9.22705
CD11c+ cell	0.12156	0.00660	5.97585	5.27798	1.82174	0.92746	0.00705	2.95962
Epithelial cell	36.08627	72.357	13.79193	8.87779	85.19184	86.09094	0.55367	0.90230
Granulocyte (CD66b)	0.32549	0.64032	0.62021	0.47487	NA	NA	0.03173	0.22463
Helper T cell (CD4)	1.50196	NA	27.55218	28.71602	0.36489	0.27415	0.19748	33.39449
Macrophage (CD68, CD163)	7.62745	2.72304	5.59780	1.61579	2.04504	2.07075	0.86401	2.40176
Monocyte (CD14)	0.18823	0.03960	2.39712	3.16719	1.02932	0.42873	2.40866	1.21679
NK cell (CD57)	0.20392	0.06601	0.28294	0.14890	0.03812	0.04083	0.12695	0.31823
PD1+ Cytotoxic T cell (CD8)	0.01960	NA	0.67001	0.42256	0.00544	NA	NA	0.35755
PD1+ Helper/Regulatory T cell (CD4+FoxP3+)	0.26666	0.01980	2.75023	2.74463	0.03812	NA	NA	9.28508
Regulatory T cell (CD4+FoxP3+)	0.18039	0.01980	1.50301	3.09475	0.05446	0.02333	NA	0.35755
Stromal cell (CD31)	40.49411	21.65561	12.4519	16.6006	8.07940	8.15469	53.2233	11.98076
T cell (CD3, CD4, CD8)	3.64705	0.25415	35.74177	36.26376	0.69983	0.57456	0.53251	44.31195
Negative	2.78039	2.13552	6.52813	3.24566	0.95580	1.54577	42.20624	2.59271

## Data Availability

Due to the involvement of patients in this study, participants were assured that raw patient data would remain confidential and would not be shared. Data not directly related to patients will be made available upon reasonable request to the corresponding author.

## Supplementary Materials

The following supporting information can be downloaded at: https://www.mdpi.com/article/10.3390/cancers18081285/s1, Figure S1: Monitoring for IFN-γ reactivity in vaccine patients. Table S1: Immune cell composition in the TME of DC vaccine patients at the time of diagnosis. Table S2: Antibody panel used for IMC staining. Table S3: DC vaccine patients’ cell phenotypes.

## Abbreviations

The following abbreviations are used in this manuscript:

alpha-dendritic cell-1	α-DC-1
antigen-presenting cells	APC
anti-programmed death-1	anti-PD-1
current Good Manufacturing Practices	cGMP
delayed-type hypersensitivity	DTH
dendritic cell	DC
enzyme-linked immunosorbent	ELISpot
formalin-fixed paraffin-embedded	FFPE
granulocyte–macrophage colony-stimulating factor	GM-CSF
high-grade serous ovarian cancer	HGSOC
imaging mass cytometry	IMC
immunohistochemistry	IHC
interferon-gamma	IFN-γ
Institutional Review Board	IRB
interleukin-4	IL-4
keyhole limpet hemocyanin	KLH
leukapheresis	LK
minimal residual disease	MRD
ovarian cancer	OC
overall survival	OS
peripheral blood mononuclear cells	PBMC
progression-free survival	PFS
region of interest	ROI
Response Evaluation Criteria in Solid Tumors	RECIST
tumor-infiltrating lymphocyte	TIL
tumor lysate	TL
tumor microenvironment	TME

## References

[1] Siegel R.L., Kratzer T.B., Wagle N.S., Sung H., Jemal A. (2026). Cancer statistics, 2026. CA Cancer J. Clin..

[2] Zheng L., Cui C., Shi O., Lu X., Li Y.K., Wang W., Li Y., Wang Q. (2020). Incidence and mortality of ovarian cancer at the global, regional, and national levels, 1990–2017. Gynecol. Oncol..

[3] Liu L., Xiong W. (2021). Effect of molecular targeted agents in chemotherapy for treating platinum-resistant recurrent ovarian cancer: A systematic review and meta-analysis. Medicine.

[4] Lheureux S., Braunstein M., Oza A.M. (2019). Epithelial ovarian cancer: Evolution of management in the era of precision medicine. CA Cancer J. Clin..

[5] Sambasivan S. (2022). Epithelial ovarian cancer: Review article. Cancer Treat. Res. Commun..

[6] Oronsky B., Ray C.M., Spira A.I., Trepel J.B., Carter C.A., Cottrill H.M. (2017). A brief review of the management of platinum-resistant-platinum-refractory ovarian cancer. Med. Oncol..

[7] Moore K.N., Angelergues A., Konecny G.E., García Y., Banerjee S., Lorusso D., Lee J.-Y., Moroney J.W., Colombo N., Roszak A. (2023). Mirvetuximab Soravtansine in FRα-Positive, Platinum-Resistant Ovarian Cancer. N. Engl. J. Med..

[8] Olawaiye A.B., Gladieff L., O’MAlley D.M., Kim J.-W., Garbaos G., Salutari V., Gilbert L., Mileshkin L., Devaux A., Hopp E. (2025). Relacorilant and nab-paclitaxel in patients with platinum-resistant ovarian cancer (ROSELLA): An open-label, randomised, controlled, phase 3 trial. Lancet.

[9] Zam W., Ali L. (2022). Immune Checkpoint Inhibitors in the Treatment of Cancer. Curr. Rev. Clin. Exp. Pharmacol..

[10] Vaddepally R.K., Kharel P., Pandey R., Garje R., Chandra A.B. (2020). Review of Indications of FDA-Approved Immune Checkpoint Inhibitors per NCCN Guidelines with the Level of Evidence. Cancers.

[11] Mathé G., Amiel J.L., Schwarzenberg L., Cattan A., Schneider M., De Vries M.J., Tubiana M., Lalanne C., Binet J.L., Papiernik M. (1965). Successful allogenic bone marrow transplantation in man: Chimerism, induced specific tolerance, and possible anti-leukemic effects. Blood.

[12] Zhu J., Yan L., Wang Q. (2021). Efficacy of PD-1/PD-L1 inhibitors in ovarian cancer: A single-arm meta-analysis. J. Ovarian Res..

[13] Dumitru A., Dobrica E.C., Croitoru A., Cretoiu S.M., Gaspar B.S. (2022). Focus on PD-1/PD-L1 as a Therapeutic Target in Ovarian Cancer. Int. J. Mol. Sci..

[14] Matulonis U.A., Shapira-Frommer R., Santin A.D., Lisyanskaya A.S., Pignata S., Vergote I., Raspagliesi F., Sonke G.S., Birrer M., Provencher D.M. (2019). Antitumor activity and safety of pembrolizumab in patients with advanced recurrent ovarian cancer: Results from the phase II KEYNOTE-100 study. Ann. Oncol..

[15] Walsh C.S., Kamrava M., Rogatko A., Kim S., Li A., Cass I., Karlan B., Rimel B.J. (2021). Phase II trial of cisplatin, gemcitabine and pembrolizumab for platinum-resistant ovarian cancer. PLoS ONE.

[16] Hamanishi J., Mandai M., Konishi I. (2016). Immune checkpoint inhibition in ovarian cancer. Int. Immunol..

[17] Zsiros E., Lynam S., Attwood K.M., Wang C., Chilakapati S., Gomez E.C., Liu S., Akers S., Lele S., Frederick P.J. (2021). Efficacy and Safety of Pembrolizumab in Combination With Bevacizumab and Oral Metronomic Cyclophosphamide in the Treatment of Recurrent Ovarian Cancer: A Phase 2 Nonrandomized Clinical Trial. JAMA Oncol..

[18] Fucikova J., Coosemans A., Orsulic S., Cibula D., Vergote I., Galluzzi L., Spisek R. (2021). Immunological configuration of ovarian carcinoma: Features and impact on disease outcome. J. Immunother. Cancer.

[19] Yang Y., Yang Y., Yang J., Zhao X., Wei X. (2020). Tumor Microenvironment in Ovarian Cancer: Function and Therapeutic Strategy. Front. Cell Dev. Biol..

[20] Colombo N., Zsiros E., Sebastianelli A., Bidzinski M., Araneda C.G., Matanes E., Hasegawa K., Kose F., Maciel M.M., Herbertson R. (2025). LBA3 Pembrolizumab vs placebo plus weekly paclitaxel ± bevacizumab in platinum-resistant recurrent ovarian cancer: Results from the randomized double-blind phase III ENGOT-ov65/KEYNOTE-B96 study. Ann. Oncol..

[21] Jin F., Xie L., Zhang H., Fan X., Tian J., Liu W., Xiao Y., Fan X. (2025). Dendritic Cells: Origin, Classification, Development, Biological Functions, and Therapeutic Potential. MedComm.

[22] Banchereau J., Briere F., Caux C., Davoust J., Lebecque S., Liu Y.J., Pulendran B., Palucka K. (2000). Immunobiology of dendritic cells. Annu. Rev. Immunol..

[23] Chen F., Hou M., Ye F., Lv W., Xie X. (2009). Ovarian cancer cells induce peripheral mature dendritic cells to differentiate into macrophagelike cells in vitro. Int. J. Gynecol. Cancer Off. J. Int. Gynecol. Cancer Soc..

[24] Scarlett U.K., Rutkowski M.R., Rauwerdink A.M., Fields J., Escovar-Fadul X., Baird J., Cubillos-Ruiz J.R., Jacobs A.C., Gonzalez J.L., Weaver J. (2012). Ovarian cancer progression is controlled by phenotypic changes in dendritic cells. J. Exp. Med..

[25] Chen J., Duan Y., Che J., Zhu J. (2024). Dysfunction of dendritic cells in tumor microenvironment and immunotherapy. Cancer Commun..

[26] Palucka K., Banchereau J. (2012). Cancer immunotherapy via dendritic cells. Nat. Rev. Cancer.

[27] Sabado R.L., Balan S., Bhardwaj N. (2017). Dendritic cell-based immunotherapy. Cell Res..

[28] Marciscano A.E., Anandasabapathy N. (2021). The role of dendritic cells in cancer and anti-tumor immunity. Semin. Immunol..

[29] Rob L., Cibula D., Knapp P., Mallmann P., Klat J., Minar L., Bartos P., Chovanec J., Valha P., Pluta M. (2022). Safety and efficacy of dendritic cell-based immunotherapy DCVAC/OvCa added to first-line chemotherapy (carboplatin plus paclitaxel) for epithelial ovarian cancer: A phase 2, open-label, multicenter, randomized trial. J. Immunother. Cancer.

[30] Borges F., Laureano R.S., Vanmeerbeek I., Sprooten J., Demeulenaere O., Govaerts J., Kinget L., Saraswat S., Beuselinck B., De Vleeschouwer S. (2024). Trial watch: Anticancer vaccination with dendritic cells. Oncoimmunology.

[31] Hernando J.J., Park T.W., Kubler K., Offergeld R., Schlebusch H., Bauknecht T. (2002). Vaccination with autologous tumour antigen-pulsed dendritic cells in advanced gynaecological malignancies: Clinical and immunological evaluation of a phase I trial. Cancer Immunol. Immunother..

[32] Tanyi J.L., Bobisse S., Ophir E., Tuyaerts S., Roberti A., Genolet R., Baumgartner P., Stevenson B.J., Iseli C., Dangaj D. (2018). Personalized cancer vaccine effectively mobilizes antitumor T cell immunity in ovarian cancer. Sci. Transl. Med..

[33] Kandalaft L.E., Chiang C.L., Tanyi J., Motz G., Balint K., Mick R., Coukos G. (2013). 3693890; A Phase I vaccine trial using dendritic cells pulsed with autologous oxidized lysate for recurrent ovarian cancer. J. Transl. Med..

[34] Zhang X., He T., Li Y., Chen L., Liu H., Wu Y., Guo H. (2020). Dendritic Cell Vaccines in Ovarian Cancer. Front. Immunol..

[35] Mailliard R.B., Wankowicz-Kalinska A., Cai Q., Wesa A., Hilkens C.M., Kapsenberg M.L., Kirkwood J.M., Storkus W.J., Kalinski P. (2004). Alpha-type-1 polarized dendritic cells: A novel immunization tool with optimized CTL-inducing activity. Cancer Res..

[36] Lambert L.A., Gibson G.R., Maloney M., Durell B., Noelle R.J., Barth R.J. (2001). Intranodal immunization with tumor lysate-pulsed dendritic cells enhances protective antitumor immunity. Cancer Res..

[37] Nestle F.O., Alijagic S., Gilliet M., Sun Y., Grabbe S., Dummer R., Burg G., Schadendorf D. (1998). Vaccination of melanoma patients with peptide- or tumor lysate-pulsed dendritic cells. Nat. Med..

[38] Dhodapkar M.V., Steinman R.M., Sapp M., Desai H., Fossella C., Krasovsky J., Donahoe S.M., Dunbar P.R., Cerundolo V., Nixon D.F. (1999). Rapid generation of broad T-cell immunity in humans after a single injection of mature dendritic cells. J. Clin. Investig..

[39] Aarntzen E.H., de Vries I.J., Goertz J.H., Beldhuis-Valkis M., Brouwers H.M., van de Rakt M.W., van der Molen R.G., Punt C.J., Adema G.J., Tacken P.J. (2012). Humoral anti-KLH responses in cancer patients treated with dendritic cell-based immunotherapy are dictated by different vaccination parameters. Cancer Immunol. Immunother..

[40] Stiff P.J., Czerlanis C., Drakes M.L. (2013). Dendritic cell immunotherapy in ovarian cancer. Expert Rev. Anticancer. Ther..

[41] Rustin G.J., Vergote I., Eisenhauer E., Pujade-Lauraine E., Quinn M., Thigpen T., du Bois A., Kristensen G., Jakobsen A., Sagae S. (2011). Definitions for response and progression in ovarian cancer clinical trials incorporating RECIST 1.1 and CA 125 agreed by the Gynecological Cancer Intergroup (GCIG). Int. J. Gynecol. Cancer.

[42] Veenstra J., Dimitrion P., Yao Y., Zhou L., Ozog D., Mi Q.S. (2021). Research Techniques Made Simple: Use of Imaging Mass Cytometry for Dermatological Research and Clinical Applications. J. Investig. Dermatol..

[43] Liu Z., Xun J., Liu S., Wang B., Zhang A., Zhang L., Wang X., Zhang Q. (2022). Imaging mass cytometry: High-dimensional and single-cell perspectives on the microenvironment of solid tumours. Prog. Biophys. Mol. Biol..

[44] The R Core Team (2025). A Language and Environment for Statistical Computing.

[45] Brandum E.P., Jørgensen A.S., Rosenkilde M.M., Hjortø G.M. (2021). Dendritic Cells and CCR7 Expression: An Important Factor for Autoimmune Diseases, Chronic Inflammation, and Cancer. Int. J. Mol. Sci..

[46] Santoiemma P.P., Powell D.J. (2015). Tumor infiltrating lymphocytes in ovarian cancer. Cancer Biol. Ther..

[47] Fridman W.H., Pagès F., Sautès-Fridman C., Galon J. (2012). The immune contexture in human tumours: Impact on clinical outcome. Nat. Rev. Cancer.

[48] Ovarian Tumor Tissue Analysis C., Goode E.L., Block M.S., Kalli K.R., Vierkant R.A., Chen W., Fogarty Z.C., Gentry-Maharaj A., Toloczko A., Hein A. (2017). Dose-Response Association of CD8^+^ Tumor-Infiltrating Lymphocytes and Survival Time in High-Grade Serous Ovarian Cancer. JAMA Oncol..

[49] Zhang L., Conejo-Garcia J., Katsaros D., Gimotty P.A., Massobrio M., Regnani G., Makrigiannakis A., Gray H., Schlienger K., Liebman M.N. (2003). Intratumoral T cells, recurrence, and survival in epithelial ovarian cancer. N. Engl. J. Med..

[50] Hwang W.T., Adams S.F., Tahirovic E., Hagemann I.S., Coukos G. (2012). Prognostic significance of tumor-infiltrating T cells in ovarian cancer: A meta-analysis. Gynecol. Oncol..

[51] Bray F., Laversanne M., Sung H., Ferlay J., Siegel R.L., Soerjomataram I., Jemal A. (2024). Global cancer statistics 2022: GLOBOCAN estimates of incidence and mortality worldwide for 36 cancers in 185 countries. CA Cancer J. Clin..

[52] Lee J.J., Foon K.A., Mailliard R.B., Muthuswamy R., Kalinski P. (2008). Type 1-polarized dendritic cells loaded with autologous tumor are a potent immunogen against chronic lymphocytic leukemia. J. Leukoc. Biol..

[53] Kantoff P.W., Higano C.S., Shore N.D., Berger E.R., Small E.J., Penson D.F., Redfern C.H., Ferrari A.C., Dreicer R., Sims R.B. (2010). Sipuleucel-T immunotherapy for castration-resistant prostate cancer. N. Engl. J. Med..

[54] Mitsuya K., Akiyama Y., Iizuka A., Miyata H., Deguchi S., Hayashi N., Maeda C., Kondou R., Kanematsu A., Watanabe K. (2020). Alpha-type-1 Polarized Dendritic Cell-based Vaccination in Newly Diagnosed High-grade Glioma: A Phase II Clinical Trial. Anticancer. Res..

