# Supplementary material for: Outcomes of an Alpha-DC-1 Dendritic Cell-Based Vaccine Clinical Trial in Patients with Low-Tumor-Burden High-Risk Ovarian Carcinoma
Source: Cancers (Basel). 2026 Apr 18;18(8):1285. doi: 10.3390/cancers18081285 (PMC13114759; doi:10.3390/cancers18081285)
Supplement: Supplementary file 1 [file cancers-18-01285-s001.zip › cancers-4211619-supplementary.pdf]

CELL TYPE	34CO	40KR	46KK	52MK	59EDC	60ML	66TS	57LMO	69NW	70VS	78LC2	79SP2	80WS	83SF	88 MLM
CD8	3, S	2	4, C	2 (L.T.)	3, C+ S	2	1, S	4	1, S (L.T.)	4	4, C	N.T.	2	2, S	4, C
CD3	2	2	4	3	4	N.D.	1	4	N.T.	4	4	0	2	2	4
FOXP3	1, S	2	2, S	2, S	1, S	1	1, S	3, S	4, S	3, S	3, S	1	1	2,S	3, S

Table S1. Immune cell composition in the TME of DC vaccine patients at the time of diagnosis. Ovarian cancer FFPE tissues were IHC stained using primary antibodies for CD3 (F7.2.38; 1:1000 dilution, Dako, Glostrup, Denmark), CD8 (C8/144B; 1: 100 dilution, Cell Marque, Rocklin, CA) and FoxP3 (236A/ E7; ab 20034, Abcam). Staining was completed using the Vector Laboratories peroxidase system and sections were counterstained in hematoxylin. Tumor infiltrating lymphocytes (TILs) were quantitated and averaged over 5-10 high power fields (h.p.f.). L.T. indicates low tumor in the section. N.T indicates no tumor seen in section. N.D. indicates not done. Pathology scoring parameters: CD3 and CD8: 0 = <1, 1 = 1 – 15, 2 = 15 – 5, 3= 25 – 50, 4 = >50 cells/ h.p.f. FoxP3: 0 = <1, 1 = 1 – 5, 2 = 5 – 15, 3= >15 – 25, 4 = >25/ h.p.f. "C" indicates a clustered pattern of TILs and "S" indicates that the cells were in a scattered pattern.

Target	Clone	Metal Tag	Dilution	Target	Clone	Metal Tag	Dilution
Collagen I	Polyclonal	89Y	100	CD57	NK/804	163Dy	100
CD20	H1	115In	100	Podoplanin	D2-40	164Dy	100
Pan-Cytokeratin	AE-1/AE-3	141Pr	500	PD-1	D4W2J	165Ho	50
CD11b	EPR1344	144Nd	100	PD-L1	73-10	166Er	50
CD163	EDHu-1	147Sm	100	B-Catenin	5H10	169Tm	200
Vimentin	D21H3	149Sm	400	CD3	D7A6E	170Er	100
Ki-67	B56	150Nd	100	Fibronectin	EPR23110-46	171Yb	500
CD31	EPR3094	151Eu	50	EpCAM	EPR20532-222	172YB	100
CD45	D9M8I	152Sm	100	CD45RO	UCHL1	173Yb	250
CD44	IM7	153Eu	50	HLA-DR	LN3	174Yb	200
CD11c	D3V1E	154Sm	100	CD14	EPR3653	175Lu	200
FoxP3	PCH101	155Gd	50	Granzyme B	EPR20129-217	176Yb	250
CD4	EPR6855	156Gd	50	DNA1	-	191Ir	400
E-Cadherin	24E10	158Gd	100	DNA2	-	193Ir	400
CD68	KP1	159Tb	100	ICSK1	-	195Pt	100
CD66b	BLR111H	160Gd	100	ICSK2	-	196Pt	100
FAP	E1V9V	161Dy	50	ICSK3	-	198Pt	100
CD8a	C8/144B	162Dy	100	aSMA	1A4	209Bi	500

Table S2. Antibody panel used for IMC staining (Standard BioTools).

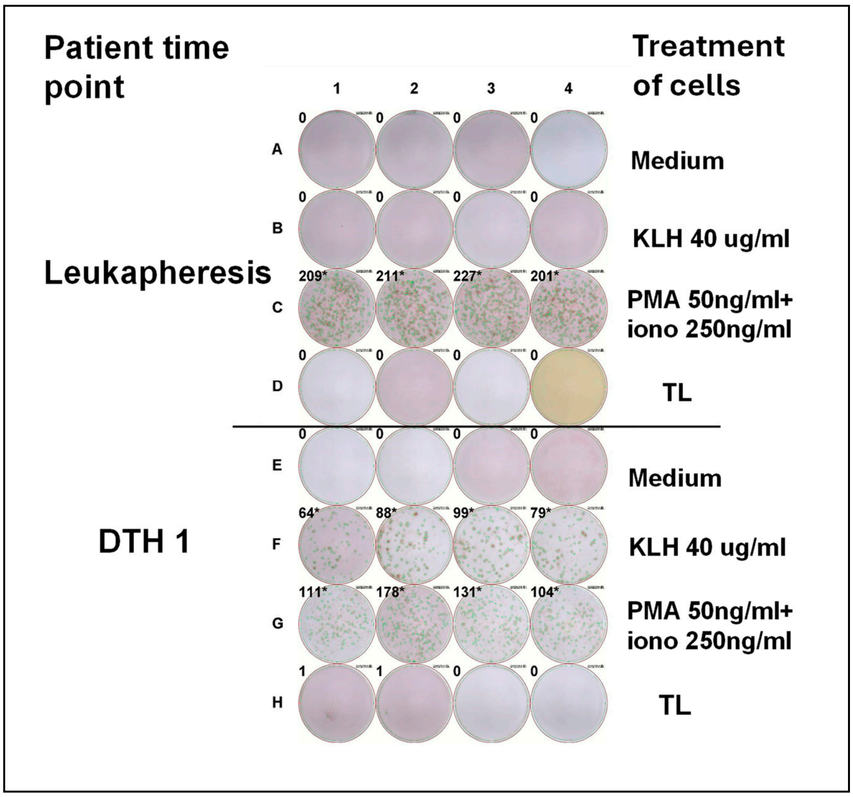

Figure S1. Monitoring for IFN-γ reactivity in vaccine patients. ELISpot assays were performed on patient PBMCs collected prior to each vaccine (LK time point) as a baseline and at DTH1 (day 61 post vaccine 1 of cycle 1) time points. PBMC were stimulated with KLH, tumor lysate (TL) or PMA and ionomycin (positive control) over 48 hours culture in multiple wells to detect IFN-γ secretion in T cells using the DIK3420-1000 kit (Mabtech). Spots were developed for 10 mins, plates washed and air dried. The number of IFN-γ secreting spots on ELISpot plates were captured on a C.T.L Cellular Technology Immunospot machine and evaluated using Immunospot 3 and 5 software, respectively. A positive response was determined as the number of IFN-γ secreting spots greater than 2 times the background spots in medium. Representative results are shown as captured for patient 57LMO.

Table S3. DC vaccine patients' cell phenotypes. PBMC were stained with monoclonal antibodies conjugated to multiple fluorochromes to identify cell subsets of T cells, CD19 (B cells), CD40 (co-stimulatory molecules), CD14 (macrophages) in patients administered DC vaccines. Staining was done in batches during the course of the study, with a normal PBMC control added each day of staining. Events were acquired on a flow cytometer and gating done in the lymphocyte or live cell region. FlowJo analysis was used to identify the percentage of cells in each subset including CD45RO memory T cells, CD8+CD28+ immunocompetent T cells and CD19+CD40+ B cells. Representative data is shown as for patient CD34CO.

Schedule	Day	CD3	CD3+CD45RO+	CD8+CD45RO+	CD8	CD8+CD28+	CD19+CD40+	CD14
	Control 9	*66*	*22.5*	*4.53*	*16.4*	*10.7*	*10.6*	*25.9*
**LK day**	**-7**	**61.6**	**40.2**	**7.21**	**15.98**	**9.96**	**8.12**	**45.2**
**Vaccine 1, cycle 1**	0	52.2	33.6	5.59	16.58	8.13	9.87	35.6
	5	64.5	38.9	5.71	16.53	9.86	12.20	27.8
	Control 9	*68.3*	*24.5*	*3.77*	*14.31*	*9.8*	*10.20*	*23*
	12	53.4	35.5	5.15	16.45	8.41	11.30	41.4
**Vaccine 2, cycle 1**	21	53.6	34	4.05	12.92	7.18	20.3	34.70
**Vaccine 3, cycle 1**	34	57.2	35.7	4.21	13.07	7.19	19.00	40.8
	Control 9	*67.8*	*23.3*	*4.78*	*15.96*	*11.1*	*9.9*	*22.6*
	41	54.5	40.6	5.26	13.89	8.66	15.30	31
	55	50.7	34.1	3.93	12.58	7.27	20.00	36.7
	63	58.3	36.5	3.88	12.07	8.08	23.00	30.1
	Control 9	*69.1*	*27.3*	*4.77*	*18.51*	*12.7*	*9.12*	*24.5*
	75	54.4	38.1	4.61	13.46	8.52	23	40.9
**Vaccine 1, cycle 2**	83	55.4	38.4	4.59	14.31	9.53	21.7	35.7
	90	54.6	36.9	4.88	14.77	8.43	16.5	36.1
	Control 9	*69.5*	*24.8*	*4.66*	*17.02*	*11.6*	*9.78*	*25.8*
**Vaccine 2, cycle 2**	97	47.1	26.8	4.54	12.64	8.97	30.5	32.6
	105	51.8	30.4	4.24	12.35	7.28	17.8	37.8
**Vaccine 3, cycle 2**	112	55.6	28.4	2.85	10.35	7.2	27.9	31.2
	Control 9	*70*	*23.5*	*4.98*	*17.46*	*12.5*	*10.2*	*25.3*
	118	53.1	33.9	4.8	12.68	7.94	23.4	35.7
	125	46.1	27.1	4.82	12.5	8.74	28.1	37.4
	132	48.9	29.7	4.8	12.18	8.04	29.7	34.2
	Control 9	*66.2*	*21.8*	*3.6*	*15*	*10.3*	*10.1*	*22.8*
	140	44	26.6	3.45	10.21	6.15	30.4	32.3
	145	42.7	28.6	2.53	8.24	5.09	32.4	33.7
**Vaccine 1, cycle 3**	163	53.7	31.7	2.96	9.93	7.12	29.4	24.1
	Control 9	68.7	20.7	3.86	16.73	11.7	10.4	21.6
	172	51.1	25.4	3.97	13.64	10.4	23.2	23.9
**Vaccine 2, cycle 3**	177	50.7	29.8	3.29	9.8	6.85	30.7	29.4
	185	49.5	26	3.57	11.2	8.54	30.8	19.8
**Vaccine 3, cycle 3**	191	57.3	37	5.34	12.62	7.78	17.5	33.2
	199	40.5	21.4	5.56	13.89	9.45	34.3	32.8
	206	41.4	21.4	4.87	13.1	9.27	39.8	28.7
	214	40.8	21.6	4.44	13	8.96	34.4	27
	Control 9	*69.9*	*21.5*	*3.41*	*15.47*	*10.2*	*10.8*	*22.9*
	220	42.4	23	3.24	10.4	7.13	30.1	45.8
	235	47.4	26.5	3.07	10.05	5.82	27.9	39.2
	Control 9	*66.9*	*20.7*	*4.78*	*18.53*	*12.9*	*10.1*	*23.2*
	339	44.8	23.9	4.39	13.36	9.11	35.2	17.1
